# When the Ends Justify the Means: Regulation of Telomere Addition at Double-Strand Breaks in Yeast

**DOI:** 10.3389/fcell.2021.655377

**Published:** 2021-03-18

**Authors:** Remington E. Hoerr, Katrina Ngo, Katherine L. Friedman

**Affiliations:** Department of Biological Sciences, Vanderbilt University, Nashville, TN, United States

**Keywords:** telomere, telomerase, *de novo* telomere addition, DNA repair, Pif1

## Abstract

Telomeres, repetitive sequences located at the ends of most eukaryotic chromosomes, provide a mechanism to replenish terminal sequences lost during DNA replication, limit nucleolytic resection, and protect chromosome ends from engaging in double-strand break (DSB) repair. The ribonucleoprotein telomerase contains an RNA subunit that serves as the template for the synthesis of telomeric DNA. While telomere elongation is typically primed by a 3′ overhang at existing chromosome ends, telomerase can act upon internal non-telomeric sequences. Such *de novo* telomere addition can be programmed (for example, during chromosome fragmentation in ciliated protozoa) or can occur spontaneously in response to a chromosome break. Telomerase action at a DSB can interfere with conservative mechanisms of DNA repair and results in loss of distal sequences but may prevent additional nucleolytic resection and/or chromosome rearrangement through formation of a functional telomere (termed “chromosome healing”). Here, we review studies of spontaneous and induced DSBs in the yeast *Saccharomyces cerevisiae* that shed light on mechanisms that negatively regulate *de novo* telomere addition, in particular how the cell prevents telomerase action at DSBs while facilitating elongation of critically short telomeres. Much of our understanding comes from the use of perfect artificial telomeric tracts to “seed” *de novo* telomere addition. However, endogenous sequences that are enriched in thymine and guanine nucleotides on one strand (TG-rich) but do not perfectly match the telomere consensus sequence can also stimulate unusually high frequencies of telomere formation following a DSB. These observations suggest that some internal sites may fully or partially escape mechanisms that normally negatively regulate *de novo* telomere addition.

## Introduction

Most linear eukaryotic chromosomes terminate in protein-bound repetitive sequences termed telomeres. Telomere sequences are highly repetitive and contain a thymine and guanine-rich (TG-rich)-rich 3′ terminating strand that extends beyond the 5′ strand to create a single-stranded (ss) overhang. Telomeres protect chromosome ends from nucleolytic resection (thereby preventing checkpoint activation) and provide a mechanism to counteract progressive loss of terminal sequences during DNA replication [reviewed in Osterhage and Friedman (2009) and Wellinger and Zakian (2012)]. Telomere maintenance is achieved by the enzyme telomerase, a reverse transcriptase that utilizes its RNA subunit as a template for telomere synthesis (Greider and Blackburn, 1987). Following extension of the 3′ overhang by telomerase, the complementary C-rich strand is generated by the lagging strand polymerase machinery (Wellinger and Zakian, 2012; Churikov et al., 2013).

In the budding yeast *Saccharomyces cerevisiae*, extension by telomerase, C-strand fill-in, and telomere protection are coordinated by the CST (Cdc13-Stn1-Ten1) complex (Churikov et al., 2013). Cdc13 binds ss telomeric repeats and interacts with the Est1 subunit of telomerase to initiate telomere extension (Pennock et al., 2001). Stn1 and Ten1 coordinate C-strand fill-in and end protection (Pennock et al., 2001; Xu et al., 2009; Ge et al., 2020). CST functions are highly coordinated in the cell cycle, and Stn1 competes with Est1 for association with Cdc13 at telomeres to prevent overextension of the 3′ end and promote 5′ C-strand fill-in (Gopalakrishnan et al., 2017). The “capping” function of the CST complex prevents excessive nucleolytic resection and the activation of DSB repair pathways at telomeres (Garvik et al., 1995). Recent evidence shows that unregulated resection ensues when telomeres undergo replication in the absence of Cdc13 function (Langston et al., 2020).

While it is important to prevent the DNA repair machinery from processing telomeres, it is equally important for cells to prevent telomerase from acting at a DSB—such events interfere with normal repair and result in loss of distal sequences. This process is termed chromosome healing (since the new telomere prevents additional nucleolytic resection), chromosome/telomere capture, or *de novo* telomere addition (dnTA). DnTA can be developmentally regulated. For example, ciliated protozoa undergo programmed mass genome fragmentation during macronuclear development where each newly formed linear fragment acquires *de novo* telomeres (Jahn and Klobutcher, 2002). In other cases, dnTA is pathogenic. In humans, multiple diseases, such as Phelan/McDermid syndrome (Bonaglia et al., 2011) and α-thalassemia (Wilkie et al., 1990), are attributed to terminal deletions *via* dnTA. Much of our knowledge on mechanisms that regulate dnTA at chromosome breaks comes from studies in budding yeast. Here, we review mechanisms in yeast that limit dnTA at DSBs and discuss the nature of chromosome sites with an unusual propensity to undergo telomere healing.

## Strategies to Study *de novo* Telomere Addition in Yeast

Two strategies are commonly used to study dnTA in yeast. In the first, cells are selected for loss of two distal, counter-selectable markers. Rare gross chromosomal rearrangement (GCR) events are recovered and analyzed to determine where the chromosome break resolved (Schmidt et al., 2006). Importantly, the location of the initiating break may not be coincident with the site of repair since resection can occur prior to resolution of the break. The broken chromosome may be stabilized by dnTA, a large internal deletion, or through translocation. GCR assays have been instrumental in the identification and characterization of *cis*- and *trans*-acting factors that promote genome stability (Chen and Kolodner, 1999; Myung et al., 2001).

The second strategy to study dnTA involves generation of an induced DSB, most predominantly using the homothallic switching (HO) endonuclease (Sugawara and Haber, 2012). Strains in which the gene encoding the HO endonuclease is controlled by an inducible promoter allow regulated generation of a DSB at any chromosome location engineered to contain the cleavage site (Nickoloff et al., 1986). When the HO site is placed distal to the last essential gene in a haploid strain, cells survive continuous expression of the nuclease by incurring a localized mutation at the HO site or by losing the chromosome terminus through translocation or dnTA (Kramer and Haber, 1993). HO cleavage adjacent to an artificial telomere “seed” sequence (typically 80 bp or longer) has been successfully and extensively exploited to study telomere elongation and capping (Diede and Gottschling, 1999; Negrini et al., 2007). Because telomeric tracts of that length are not present in the yeast genome outside of sub-telomeric regions, we concentrate here on experiments in which exogenous seed sequences are either lacking or short enough to mimic endogenous sites.

**FIGURE 1:**
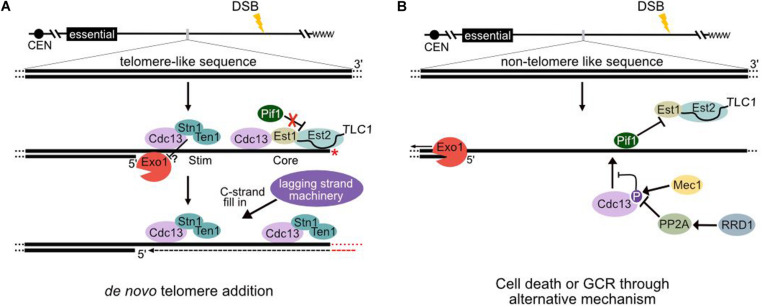
Models of telomerase regulation at a resecting break in the presence and absence of telomere-like sequences. **(A)** Regulation of telomerase at endogenous hotspots of *de novo* telomere addition [Sites of Repair-associated Telomere Addition (SiRTAs)]. Following induction of a double-strand break (DSB), the MRX complex (Mre11–Xrs2–Rad50) along with Sae2 initiates 5’ end resection. Multiple nucleases act at DSBs, but extensive resection requires the exonuclease Exo1 and helicase Sgs1 (Gravel et al., 2008; Mimitou and Symington, 2008; Zhu et al., 2008). The resulting generation of single-stranded DNA (ssDNA) triggers a checkpoint kinase cascade and cell cycle arrest (Villa et al., 2016). Following resection through the TG-rich sequences, Cdc13 binds to a “Core” sequence and recruits telomerase through interactions with Est1. Cdc13, in complex with Stn1 and Ten1 [likely as a hexamer (Ge et al., 2020)], also binds to a proximal “Stim” sequence to prevent further 5′ resection. The limited generation of ssDNA inhibits Pif1 loading and removal of telomerase (see text). While both the Stim and Core sequences are necessary to stimulate *de novo* telomere addition, it is unclear whether Cdc13 complexes bound to each are functionally distinct (as depicted here). Telomerase must access a 3′ terminus, which is generated through an unknown mechanism to prime telomere synthesis (depicted by a red *). Following *de novo* telomere addition by telomerase, the CST complex recruits the lagging strand machinery for C-strand fill-in (see text). If the site of telomere addition is oriented correctly relative to the centromere, the resulting product is a stable truncated chromosome. **(B)** Regulation of telomerase at sequences lacking extensive TG-rich sequences. In the absence of DSB repair, 5’ resection proceeds unimpeded. Phosphorylation of Cdc13 at serine 306 by Mec1 inhibits Cdc13 accumulation at TG_1__–__3_ sequences less than 11 bases (Zhang and Durocher, 2010). Pph3 phosphatase (in a manner requiring the activator Rrd1) counteracts Cdc13 phosphorylation (Zhang and Durocher, 2010), but Pif1 binds and inhibits telomerase action to strongly repress *de novo* telomere addition (Schulz and Zakian, 1994; Boulé et al., 2005; Li et al., 2014).

The HO cleavage system has an advantage over the GCR assay because the site of the initiating break is known. Even in the absence of a seed sequence, dnTA events can occur at the HO site, but they are also observed many kilobases internal, implying that telomerase can act after extensive 5′ end resection (Kramer and Haber, 1993; Mangahas et al., 2001; Obodo et al., 2016). Such events require removal of the overhanging strand, since telomerase must access a 3′ terminus for nucleotide addition (Kramer and Haber, 1993). Indeed, the 3′ overhang is quite stable since single-strand annealing occurs with high efficiency between one sequence immediately adjacent to the cleavage site and a homologous sequence up to 25 kb away (Vaze et al., 2002). The DSB-proximal sequence must persist in the 3′ overhang for many hours before the more distal sequence becomes ss (resection proceeds ∼4 kb per hour) (Fishman-Lobell et al., 1992). Whether loss of the overhang is stochastic or requires a specific endonuclease (perhaps associated with telomerase) is unknown. Regardless, this step must be considered in models of dnTA (Figure 1A).

## Regulation of *de novo* Telomere Addition

Given the potential for telomerase to compete with the DNA repair machinery at DSBs, it is not surprising that multiple mechanisms inhibit dnTA. These mechanisms fall into two classes: (1) mechanisms that spatially or temporally separate telomerase from DSBs and (2) mechanisms that alter the action of telomerase at a DSB. Examples of the first class include observations that telomerase is sequestered in the nucleolus in response to DSBs (Ouenzar et al., 2017) and that nuclear retention of Cdc13 requires association with DNA (most predominantly at telomeres), a property that may limit the concentration of free Cdc13 (Mersaoui et al., 2018). Here, we concentrate on the second class of mechanisms whereby telomerase action at a DSB is distinguished from its action at a telomere. In response to DNA damage, at least two proteins (Cdc13 and Pif1) are phosphorylated to reduce the probability of dnTA (Makovets and Blackburn, 2009; Zhang and Durocher, 2010). These mechanisms are additive, with both contributing to the extremely low rate of dnTA at most sequences (Zhang and Durocher, 2010).

### Mec1-Mediated Phosphorylation of Cdc13

Mec1, the yeast ortholog of the ATM and Rad3-related (ATR) kinase, directly phosphorylates Cdc13 at serine 306, thereby preventing the accumulation of Cdc13 at DSBs (Zhang and Durocher, 2010). Mec1 action is opposed by the Pph3 phosphatase in a manner requiring the activator Rrd1 (Figure 1B). Remarkably, deletion of *RRD1* eliminates dnTA at TG tracts of fewer than 11 bp, consistent with a requirement for Cdc13 association at such sequences. While Mec1-dependent phosphorylation of Cdc13 is detected in response to DNA damage, bulk levels of phosphorylated Cdc13 do not increase upon deletion of *PPH3* or *RRD1*, suggesting that dephosphorylation may specifically occur at DSBs. Consistent with this idea, Pph3 accumulates at HO-induced breaks (Zhang and Durocher, 2010).

The loss of dnTA events at sequences with fewer than 11 TG_1__–__3_ nucleotides is puzzling because Cdc13 binding requires 11 bases of TG-rich ssDNA. How can phosphorylation of Cdc13 influence its association with a sequence to which it is not predicted to directly bind? One possibility is that Cdc13 associates, albeit with lower affinity, to shorter TG tracts. While several positions of the 11-base Cdc13 binding site are critical (G1, G3, and T4), single mutations are tolerated in the rest of the binding site with minimal consequences for affinity (Eldridge et al., 2006; Lewis et al., 2014). Cdc13 associates with a resecting chromosome break even in regions where “ideal” Cdc13 binding sites are not present (Oza et al., 2009), suggesting that Cdc13 binds with low affinity at multiple sites or that other interactions facilitate association with ssDNA. For example, proteins such as RPA and Rad51 influence the recruitment of Cdc13 with DNA ends and the outcome of DNA repair (Epum et al., 2020).

### Pif1 as a Negative Regulator of *de novo* Telomere Addition

Pif1 is a 5′–3′ helicase with roles in telomere length regulation, Okazaki fragment processing, unwinding of G-quadruplex structures, DNA repair, disassembly of stalled replication complexes, and 5′ end resection (reviewed in Dewar and Lydall, 2012; Chung, 2014; Muellner and Schmidt, 2020). Pif1 also facilitates mitochondrial DNA replication; yeast without Pif1 are respiration incompetent (Foury and Kolodynski, 1983). The *pif1-m2* allele, which lacks the nuclear localization sequence, retains mitochondrial function but causes telomere overlengthening of ∼100 bp and increases the association of telomerase with telomeres (Schulz and Zakian, 1994; Boulé et al., 2005). *In vitro*, Pif1 preferentially unwinds DNA/RNA duplexes (Boulé and Zakian, 2007), suggesting that Pif1 removes telomerase from the telomere. Indeed, yeast telomerase is largely non-processive *in vitro* and remains bound to the primer following synthesis of a single telomeric repeat, but addition of Pif1 allows further rounds of elongation by facilitating telomerase release (Boulé et al., 2005). *In vivo*, limiting concentrations of telomerase [fewer than one telomerase complex per telomere (Mozdy and Cech, 2006)] may mean that telomerase released by Pif1 action is unlikely to result in additional telomere elongation. While Pif1 preferentially binds long telomeres *in vivo* (Phillips et al., 2015), experiments analyzing telomere addition in a single cell cycle are consistent with Pif1 action independent of telomere length, suggesting that enrichment at longer telomeres may reflect roles of Pif1 during replication (Stinus et al., 2018).

Pif1 also inhibits dnTA at DSBs. In strains lacking nuclear Pif1, telomere addition frequencies are elevated in response to spontaneous breaks and after induction of HO cleavage (200- to 1,000-fold, depending on the allele and assay) (Schulz and Zakian, 1994; Myung et al., 2001). Remarkably, roles of Pif1 at endogenous telomeres and in response to DSBs are genetically separable. Pif1 is phosphorylated in a Mec1-Rad53-Dun1-dependent manner following DNA damage, and a variant that cannot be phosphorylated at key residues (Pif1-4A) maintains normal telomere length but cannot repress dnTA despite associating at normal (or increased) levels with DSBs (Makovets and Blackburn, 2009). How phosphorylation alters Pif1 activity is unclear.

Using TG_1__–__3_ sequences of varying lengths integrated adjacent to an HO cleavage site, the Durocher lab systematically probed how Pif1 function is influenced by the telomeric character of a DSB (Strecker et al., 2017). With TG_1__–__3_ seeds of ≥34 bp, telomere addition to the broken end is observed in bulk culture and nearly 100% of cells survive HO cleavage, even in a strain expressing wild-type Pif1. In contrast, below this threshold, telomere addition is strongly reduced by the presence of Pif1. A phospho-mimetic allele of Pif1 (*pif1-4D*) does not affect the threshold, suggesting that phosphorylation cannot account for this distinction (Strecker et al., 2017). An exhaustive analysis uncovered Cdc13 as a mediator of differential Pif1 action on TG_1__–__3_ tracts of differing lengths. Cdc13 variants predicted to reduce interaction with Est1 or decrease DNA binding increased the threshold of TG_1__–__3_ sequence required for resistance to Pif1 negative regulation. These results suggest that the difference between a DSB and a short telomere is dictated by levels of Cdc13 association/function (Strecker et al., 2017). Interestingly, Hiraga and Sugimoto report that a telomeric seed sequence of 22 bp supports robust telomere addition and>90% survival following HO cleavage (Hirano and Sugimoto, 2007). Neither group directly assessed the capacity of Cdc13 to bind the seed sequence *in vitro*, so the difference in threshold may be explained by differential affinity of Cdc13 for the sequences tested.

## Endogenous Sequences That Stimulate *de novo* Telomere Addition

The observations of multiple independent dnTA events at specific genomic sites in Phelan/McDermid syndrome (Bonaglia et al., 2011) and α-thalassemia (Wilkie et al., 1990) suggest that certain sequences are prone to telomere addition. Hotspots of dnTA in yeast were first reported by the Zakian laboratory (Mangahas et al., 2001) as sites of recurrent chromosome truncation following an induced DSB, in one case as far as 50 kb internal to the cleavage site. These events occurred in the absence of *RAD52*, ruling out acquisition of telomeric repeats through recombinational repair. Both hotspots contained sequence tracts with similarity to the TG_1__–__3_ repeats of yeast telomeres, but surprisingly, the new telomeres were added to very short TG sequences located 37–49 bp distal to the longer TG-rich tracts (Mangahas et al., 2001).

TG-rich sequences have been observed to enhance telomerase action at a distance in other contexts. When linear plasmids terminating in repeats of the ciliate telomere sequence (TTGGGG) were transformed into yeast, addition of yeast TG_1__–__3_ sequences occurred within bacterial sequences retained as part of the cloning strategy (Murray et al., 1988). Ciliate telomere sequences integrated into the yeast chromosome 1–10 kb proximal to an HO cleavage site stimulated dnTA at TG tracts of 2–13 nucleotides located distal to the ciliate sequences (Kramer and Haber, 1993). Finally, when an 80-bp telomeric “seed” was integrated proximal to an HO site, telomere addition most frequently occurred directly on the TGTT-3′ overhang of the cleavage site, despite being separated from the seed sequence by non-telomeric DNA (Bairley et al., 2011). These results contradict the idea of a telomere-like sequence that acts solely by providing complementarity to the telomerase RNA and suggest that such sequences enhance the probability of telomere addition at nearby sites.

More recently, a total of seven additional hotspots of dnTA have been identified in yeast. Induction of HO cleavage at least 2 kb distal to these sequences results in telomere addition within the hotspot at a frequency ∼200-fold higher than in neighboring sequences, ruling out a model in which telomere addition at the hotspot is a simple consequence of chromosome fragility (Obodo et al., 2016; Ngo et al., 2020). We call such endogenous sequences Sites of Repair-associated Telomere Addition, or SiRTAs. SiRTAs on chromosomes 5 and 9 contain a ∼10–20-bp “Core” sequence that is the direct target of telomere addition and a similarly sized “Stim” sequence (located ∼20–30-bp centromere proximal to the Core) that, while rarely the site of telomere addition, strongly enhances the frequency of dnTA (Obodo et al., 2016). Therefore, as in the other examples outlined above, telomere addition occurs distal to a stimulating sequence.

Several lines of evidence argue that enhancement of dnTA by the Stim requires its ability to recruit Cdc13 to the resecting break. *In vitro*, Stim sequences from chromosomes 5 and 9 bind Cdc13 (Obodo et al., 2016). Mutations that eliminate Cdc13 binding reduce the frequency of dnTA, while mutations that improve Cdc13 affinity increase telomere addition. Replacement of the Stim sequence with the Gal4 upstream activating sequence reduces dnTA, but SiRTA activity is restored by expression of a fusion between Cdc13 and the Gal4 DNA-binding domain (Obodo et al., 2016; Epum et al., 2020). In contrast, similar artificial recruitment of the double-stranded telomeric DNA-binding protein Rap1 has no effect. These results are consistent with a model in which resection of the 5′ strand allows Cdc13 to bind the Stim sequence (and likely also the Core), thereby facilitating telomerase recruitment (Figure 1A).

## Do “Hotspots” of *de novo* Telomere Addition Escape Negative Regulation?

What accounts for the high frequency of dnTA at SiRTAs relative to other sequences? One intriguing possibility is that SiRTAs may escape, fully or partially, the negative regulatory pathways described above. To date, two genetic conditions have been identified that distinguish a SiRTA from other sequences. At very short TG tracts (≤4 bp), the Ku80 protein, a component of the Ku heterodimer that binds DSBs and is required for non-homologous end joining, promotes dnTA through interaction with the RNA component of telomerase. In the absence of the Yku80–telomerase RNA interaction, nearly all dnTA events identified by GCR assay are within the chromosome 5 SiRTA described above, highlighting that SiRTAs utilize a pathway independent of this association (Stellwagen et al., 2003). The SiRTA on chromosome 5 is also resistant to Mec1-mediated negative regulation of Cdc13 likely because the Core region contains sufficient imperfect TG_1__–__3_ sequences to exceed the 11-bp threshold of this regulation (Zhang and Durocher, 2010). However, resistance to Mec1 negative regulation alone does not explain the requirement for Cdc13 binding at the “Stim” sequence located 30–40 bp upstream of the site at which telomerase ultimately acts.

Insight may come from considering the mechanism(s) through which Pif1 regulates dnTA. Mutations reducing the Cdc13–Est1 interaction increase the TG_1__–__3_ threshold required for Pif1 resistance, consistent with a simple competition between Cdc13-mediated recruitment and Pif1-mediated removal of telomerase. The Durocher lab disfavors this model, since artificial recruitment of telomerase (by fusion of Cdc13 to Est1 or Est2) does not render a short (18-bp) TG_1__–__3_ tract resistant to Pif1 (Strecker et al., 2017). However, Hirano and Sugimoto (2007) report that fusion of Cdc13 and Est1 allows > 80% survival following DSB induction adjacent to an 11-bp TG_1__–__3_ sequence. In the latter experiment, the distal side of the break was capped by 81 bp of TG_1__–__3_ sequence, a situation predicted to attenuate the checkpoint response (Hirano and Sugimoto, 2007). Nevertheless, these contradictory results open the possibility that other aspects of Cdc13 function contribute to Pif1 resistance.

Pif1 enhances resection at uncapped telomeres (telomeres lacking Cdc13) in conjunction with Exonuclease 1 (Exo1) (Dewar and Lydall, 2010). Strecker et al. (2017) suggest that Cdc13 association with the TG_34_ tract (likely in complex with Stn1 and Ten1) is sufficient to block resection, while the TG_18_ tract is subjected to resection in a manner dependent (perhaps indirectly) on Pif1. Since Cdc13 binding and dnTA increase when resection is inhibited (Chung et al., 2010; Lydeard et al., 2010), disparities in the sensitivity to Pif1-mediated resection might explain the difference between a DSB (TG_18_) and short telomere (TG_34_).

We favor an alternative model that also invokes the role of CST in preventing 5′ end resection but posits a canonical role of Pif1 in removing telomerase from the DSB. As a 5′–3′ helicase, Pif1 must bind internal to the chromosome terminus to dissociate telomerase (Boulé and Zakian, 2007). *In vitro*, Pif1 requires an ss gap of ≥56 bases to dissociate telomerase and longer gaps facilitate more efficient removal (Li et al., 2014). If TG_34_ binds sufficient Cdc13 to inhibit 5′ end resection (as observed for TG_81_ repeats), insufficient ssDNA may be generated for Pif1 loading. In contrast, regions with little or no ability to bind Cdc13 would be rapidly resected, facilitating Pif1 association (Figure 1). Indeed, a 22-bp TG_1__–__3_ sequence that supports robust telomere addition and cell survival in a *PIF1* background substantially reduces 5′ end resection (Hirano and Sugimoto, 2007). Forced recruitment of Stn1 by fusion to Cdc13 is insufficient to promote telomere addition or prevent resection when the TG_1__–__3_ seed is only 11 bp (Hirano and Sugimoto, 2007), consistent with a requirement for multiple CST complexes to achieve this effect.

Intriguingly, this latter model could explain the role of the SiRTA Stim. Association of the Stim with Cdc13 may limit continued resection, thereby protecting the double-strand break from Pif1 loading. The location of the proximal enhancing sequence relative to the site of dnTA (≤50 bp) correlates well with the minimal region required for Pif1 loading (Li et al., 2014; Obodo et al., 2016). Furthermore, deletion of sequences between the Stim and Core dramatically increases dnTA (Obodo et al., 2016). Association of Cdc13 with the ssDNA produced during resection may also affect the ability of Pif1 to bind or translocate.

## Concluding Remarks

Much progress has been made in understanding how endogenous TG-rich sequences stimulate *de novo* telomere addition, but outstanding questions remain. Future studies must address whether the effects of Cdc13 binding on Pif1 function and/or 5′ end resection at sequences immediately adjacent to an HO cleavage site (described above) are similar at SiRTAs, where Cdc13 binding sites are revealed only after significant and ongoing resection. Likewise, while the role of Cdc13 at SiRTAs is well established, how Cdc13 associates with noncanonical sequences during resection and whether such binding is affected by association with binding partners must be addressed. SiRTAs may provide a “back-up” mechanism to facilitate chromosome healing by telomerase when other pathways have failed. Tests of this hypothesis will require genome-wide identification of SiRTAs and analysis of their evolutionary conservation. While the identity of the human ortholog of Cdc13 remains unclear (Ge et al., 2020; Lim et al., 2020), results described here in yeast raise the interesting possibility that recurrent sites of *de novo* telomere addition observed in some disease states may require the human Ctc1/Stn1/Ten1 complex (Stewart et al., 2018) and/or Pot1 (Smith et al., 2020), a telomeric ss binding protein that, like yeast Cdc13, plays roles in both end protection and telomerase recruitment.

## Author Contributions

RH, KN, and KF wrote the manuscript. RH designed the graphics. All authors contributed to the article and approved the submitted version.

## Conflict of Interest

The authors declare that the research was conducted in the absence of any commercial or financial relationships that could be construed as a potential conflict of interest.

## References

[B1] Bairley R. C. B. Guillaume G. Vega L. R. Friedman K. L. (2011). A mutation in the catalytic subunit of yeast telomerase alters primer-template alignment while promoting processivity and protein-DNA binding. *J. Cell Sci.* 124 (Pt 24) 4241–4252. 10.1242/jcs.090761 22193961 PMC4074303

[B2] Bonaglia M. C. Giorda R. Beri S. De Agostini C. Novara F. Fichera M. (2011). Molecular mechanisms generating and stabilizing terminal 22q13 deletions in 44 subjects with Phelan/McDermid syndrome. *PLoS Genet.* 7:e1002173. 10.1371/journal.pgen.1002173 21779178 PMC3136441

[B3] Boulé J. B. Vega L. R. Zakian V. A. (2005). The yeast Pif1p helicase removes telomerase from telomeric DNA. *Nature* 438 57–61. 10.1038/nature04091 16121131

[B4] Boulé J. B. Zakian V. A. (2007). The yeast Pif1p DNA helicase preferentially unwinds RNA-DNA substrates. *Nucleic Acids Res.* 35 5809–5818. 10.1093/nar/gkm613 17720711 PMC2034482

[B5] Chen C. Kolodner R. D. (1999). Gross chromosomal rearrangements in *Saccharomyces cerevisiae* replication and recombination defective mutants. *Nat. Genet.* 23 81–85. 10.1038/12687 10471504

[B6] Chung W. H. (2014). To peep into Pif1 helicase: multifaceted all the way from genome stability to repair-associated DNA synthesis. *J. Microbiol.* 52 89–98. 10.1007/s12275-014-3524-3 24500472

[B7] Chung W. H. Zhu Z. Papusha A. Malkova A. Ira G. (2010). Defective resection at DNA double-strand breaks leads to *de novo* telomere formation and enhances gene targeting. *PLoS Genet.* 6:e1000948. 10.1371/journal.pgen.1000948 20485519 PMC2869328

[B8] Churikov D. Corda Y. Luciano P. Géli V. (2013). Cdc13 at a crossroads of telomerase action. *Front. Oncol.* 3:39. 10.3389/fonc.2013.00039 23450759 PMC3584321

[B9] Dewar J. M. Lydall D. (2010). Pif1- and Exo1-dependent nucleases coordinate checkpoint activation following telomere uncapping. *EMBO J.* 29 4020–4034. 10.1038/emboj.2010.267 21045806 PMC3020640

[B10] Dewar J. M. Lydall D. (2012). Similarities and differences between “uncapped” telomeres and DNA double-strand breaks. *Chromosoma* 121 117–130. 10.1007/s00412-011-0357-2 22203190

[B11] Diede S. J. Gottschling D. E. (1999). Telomerase-mediated telomere addition in vivo requires DNA primase and DNA polymerases alpha and delta. *Cell* 99 723–733. 10.1016/s0092-8674(00)81670-0 10619426

[B12] Eldridge A. M. Halsey W. A. Wuttke D. S. (2006). Identification of the determinants for the specific recognition of single-strand telomeric DNA by Cdc13. *Biochemistry* 45 871–879. 10.1021/bi0512703 16411763 PMC3514546

[B13] Epum E. A. Mohan M. J. Ruppe N. P. Friedman K. L. (2020). Interaction of yeast Rad51 and Rad52 relieves Rad52-mediated inhibition of *de novo* telomere addition. *PLoS Genet.* 16:e1008608. 10.1371/journal.pgen.1008608 32012161 PMC7018233

[B14] Fishman-Lobell J. Rudin N. Haber J. E. (1992). Two alternative pathways of double-strand break repair that are kinetically separable and independently modulated. *Mol. Cell. Biol.* 12 1292–1303. 10.1128/mcb.12.3.1292 1545810 PMC369562

[B15] Foury F. Kolodynski J. (1983). Pif mutation blocks recombination between mitochondrial rho+ and rho- genomes having tandemly arrayed repeat units in *Saccharomyces cerevisiae*. *Proc. Natl. Acad. Sci. U.S.A.* 80 5345–5349. 10.1073/pnas.80.17.5345 6310571 PMC384252

[B16] Garvik B. Carson M. Hartwell L. (1995). Single-stranded DNA arising at telomeres in cdc13 mutants may constitute a specific signal for the *RAD9* checkpoint. *Mol. Cell. Biol.* 15 6128–6138. 10.1128/mcb.15.11.6128 7565765 PMC230864

[B17] Ge Y. Wu Z. Chen H. Zhong Q. Shi S. Li G. (2020). Structural insights into telomere protection and homeostasis regulation by yeast CST complex. *Nat. Struct. Mol. Biol.* 27 752–762. 10.1038/s41594-020-0459-8 32661422

[B18] Gopalakrishnan V. Tan C. R. Li S. (2017). Sequential phosphorylation of CST subunits by different cyclin-Cdk1 complexes orchestrate telomere replication. *Cell Cycle* 16 1271–1287. 10.1080/15384101.2017.1312235 28650257 PMC5531626

[B19] Gravel S. Chapman J. R. Magill C. Jackson S. P. (2008). DNA helicases Sgs1 and BLM promote DNA double-strand break resection. *Genes Dev.* 22 2767–2772. 10.1101/gad.503108 18923075 PMC2569880

[B20] Greider C. W. Blackburn E. H. (1987). The telomere terminal transferase of *Tetrahymena* is a ribonucleoprotein enzyme with two kinds of primer specificity. *Cell* 51 887–898. 10.1016/0092-8674(87)90576-9 3319189

[B21] Hirano Y. Sugimoto K. (2007). Cdc13 telomere capping decreases Mec1 association but does not affect Tel1 association with DNA ends. *Mol. Biol. Cell* 18 2026–2036. 10.1091/mbc.E06-12-1074 17377065 PMC1877102

[B22] Jahn C. L. Klobutcher L. A. (2002). Genome remodeling in ciliated protozoa. *Annu. Rev. Microbiol.* 56 489–520. 10.1146/annurev.micro.56.012302.160916 12142486

[B23] Kramer K. M. Haber J. E. (1993). New telomeres in yeast are initiated with a highly selected subset of TG1-3 repeats. *Genes Dev.* 7 2345–2356. 10.1101/gad.7.12a.2345 8253381

[B24] Langston R. E. Palazzola D. Bonnell E. Wellinger R. J. Weinert T. (2020). Loss of Cdc13 causes genome instability by a deficiency in replication-dependent telomere capping. *PLoS Genet.* 16:e1008733. 10.1371/journal.pgen.1008733 32287268 PMC7205313

[B25] Lewis K. A. Pfaff D. A. Earley J. N. Altschuler S. E. Wuttke D. S. (2014). The tenacious recognition of yeast telomere sequence by Cdc13 is fully exerted by a single OB-fold domain. *Nucleic Acids Res.* 42 475–484. 10.1093/nar/gkt843 24057216 PMC3874162

[B26] Li J. R. Yu T. Y. Chien I. C. Lu C. Y. Lin J. J. Li H. W. (2014). Pif1 regulates telomere length by preferentially removing telomerase from long telomere ends. *Nucleic Acids Res.* 42 8527–8536. 10.1093/nar/gku541 24981509 PMC4117769

[B27] Lim C. J. Barbour A. T. Zaug A. J. Goodrich K. J. McKay A. E. Wuttke D. S. (2020). The structure of human CST reveals a decameric assembly bound to telomeric DNA. *Science* 368 1081–1085. 10.1126/science.aaz9649 32499435 PMC7559292

[B28] Lydeard J. R. Lipkin-Moore Z. Jain S. Eapen V. V. Haber J. E. (2010). Sgs1 and Exo1 redundantly inhibit break-induced replication and *de novo* telomere addition at broken chromosome ends. *PLoS Genet.* 6:25. 10.1371/journal.pgen.1000973 20523895 PMC2877739

[B29] Makovets S. Blackburn E. H. (2009). DNA damage signalling prevents deleterious telomere addition at DNA breaks. *Nat. Cell Biol.* 11 1383–1386. 10.1038/ncb1985 19838171 PMC2806817

[B30] Mangahas J. L. Alexander M. K. Sandell L. L. Zakian V. A. (2001). Repair of chromosome ends after telomere loss in *Saccharomyces*. *Mol. Biol. Cell* 12 4078–4089. 10.1091/mbc.12.12.4078 11739802 PMC60777

[B31] Mersaoui S. Y. Bonnell E. Wellinger R. J. (2018). Nuclear import of Cdc13 limits chromosomal capping. *Nucleic Acids Res.* 46 2975–2989. 10.1093/nar/gky085 29432594 PMC5887288

[B32] Mimitou E. P. Symington L. S. (2008). Sae2. Exo1 and Sgs1 collaborate in DNA double-strand break processing. *Nature* 455 770–774. 10.1038/nature07312 18806779 PMC3818707

[B33] Mozdy A. D. Cech T. R. (2006). Low abundance of telomerase in yeast: implications for telomerase haploinsufficiency. *RNA* 12 1721–1737. 10.1261/rna.134706 16894218 PMC1557690

[B34] Muellner J. Schmidt K. H. (2020). Yeast genome maintenance by the multifunctional *PIF1* DNA helicase family. *Genes (Basel).* 11:224. 10.3390/genes11020224 32093266 PMC7073672

[B35] Murray A. W. Claus T. E. Szostak J. W. (1988). Characterization of two telomeric DNA processing reactions in *Saccharomyces cerevisiae*. *Mol. Cell. Biol.* 8 4642–4650. 10.1128/mcb.8.11.4642 3062364 PMC365553

[B36] Myung K. Chen C. Kolodner R. D. (2001). Multiple pathways cooperate in the suppression of genome instability in *Saccharomyces cerevisiae*. *Nature* 411 1073–1076. 10.1038/35082608 11429610

[B37] Negrini S. Ribaud V. Bianchi A. Shore D. (2007). DNA breaks are masked by multiple Rap1 binding in yeast: Implications for telomere capping and telomerase regulation. *Genes Dev.* 21 292–302. 10.1101/gad.400907 17289918 PMC1785115

[B38] Ngo K. Epum E. A. Friedman K. L. (2020). Emerging non-canonical roles for the Rad51–Rad52 interaction in response to double-strand breaks in yeast. *Curr. Genet.* 66 917–926. 10.1007/s00294-020-01081-z 32399607 PMC7492393

[B39] Nickoloff J. A. Chen E. Y. Heffron F. (1986). A 24-base-pair DNA sequence from the MAT locus stimulates intergenic recombination in yeast. *Proc. Natl. Acad. Sci. U.S.A.* 83 7831–7835. 10.1073/pnas.83.20.7831 3020559 PMC386816

[B40] Obodo U. C. Epum E. A. Platts M. H. Seloff J. Dahlson N. A. Velkovsky S. M. (2016). Endogenous hot spots of *de novo* telomere addition in the yeast genome contain proximal enhancers that bind Cdc13. *Mol. Cell. Biol.* 36 1750–1763. 10.1128/mcb.00095-16 27044869 PMC4907100

[B41] Osterhage J. L. Friedman K. L. (2009). Chromosome end maintenance by telomerase. *J. Biol. Chem.* 284 16061–16065. 10.1074/jbc.R900011200 19286666 PMC2713563

[B42] Ouenzar F. Lalonde M. Laprade H. Morin G. Gallardo F. Tremblay-Belzile S. (2017). Cell cycle-dependent spatial segregation of telomerase from sites of DNA damage. *J. Cell Biol.* 216 2355–2371. 10.1083/jcb.201610071 28637749 PMC5551704

[B43] Oza P. Jaspersen S. L. Miele A. Dekker J. Peterson C. L. (2009). Mechanisms that regulate localization of a DNA double-strand break to the nuclear periphery. *Genes Dev.* 23 912–927. 10.1101/gad.1782209 19390086 PMC2675867

[B44] Pennock E. Buckley K. Lundblad V. (2001). Cdc13 delivers separate complexes to the telomere for end protection and replication. *Cell* 104 387–396. 10.1016/s0092-8674(01)00226-4 11239396

[B45] Phillips J. A. Chan A. Paeschke K. Zakian V. A. (2015). The Pif1 helicase, a negative regulator of telomerase, acts preferentially at long telomeres. *PLoS Genet.* 11:e1005186. 10.1371/journal.pgen.1005186 25906395 PMC4408051

[B46] Schmidt K. H. Pennaneach V. Putnam C. D. Kolodner R. D. (2006). Analysis of gross-chromosomal rearrangements in *Saccharomyces cerevisiae*. *Methods Enzymol.* 409 462–476. 10.1016/S0076-6879(05)09027-0 16793418

[B47] Schulz V. P. Zakian V. A. (1994). The *Saccharomyces PIF1* DNA helicase inhibits telomere elongation and *de novo* telomere formation. *Cell* 76 145–155. 10.1016/0092-8674(94)90179-1 8287473

[B48] Smith E. M. Pendlebury D. F. Nandakumar J. (2020). Structural biology of telomeres and telomerase. *Cell. Mol. Life Sci.* 77 61–79. 10.1007/s00018-019-03369-x 31728577 PMC6986361

[B49] Stellwagen A. E. Haimberger Z. W. Veatch J. R. Gottschling D. E. (2003). Ku interacts with telomerase RNA to promote telomere addition at native and broken chromosome ends. *Genes Dev.* 17 2384–2395. 10.1101/gad.1125903 12975323 PMC218076

[B50] Stewart J. A. Wang Y. Ackerson S. M. Schuck P. L. (2018). Emerging roles of CST in maintaining genome stability and human disease. *Front. Biosci.* 23:1564–1586. 10.2741/4661 29293451 PMC6309963

[B51] Stinus S. Paeschke K. Chang M. (2018). Telomerase regulation by the Pif1 helicase: a length-dependent effect? *Curr. Genet.* 64 509–513. 10.1007/s00294-017-0768-6 29052759 PMC5851688

[B52] Strecker J. Stinus S. Caballero M. P. Szilard R. K. Chang M. Durocher D. (2017). A sharp Pif1-dependent threshold separates DNA double-strand breaks from critically short telomeres. *Elife* 6:e23783. 10.7554/eLife.23783 28826474 PMC5595431

[B53] Sugawara N. Haber J. E. (2012). Monitoring DNA recombination initiated by HO endonuclease. *Methods Mol. Biol.* 920 349–370. 10.1007/978-1-61779-998-3_25 22941616

[B54] Vaze M. B. Pellicioli A. Lee S. E. Ira G. Liberi G. Arbel-Eden A. (2002). Recovery from checkpoint-mediated arrest after repair of a double-strand break requires Srs2 helicase. *Mol. Cell* 10 373–385. 10.1016/S1097-2765(02)00593-2 12191482

[B55] Villa M. Cassani C. Gobbini E. Bonetti D. Longhese M. P. (2016). Coupling end resection with the checkpoint response at DNA double-strand breaks. *Cell. Mol. Life Sci.* 73 3655–3663. 10.1007/s00018-016-2262-6 27141941 PMC11108263

[B56] Wellinger R. J. Zakian V. A. (2012). Everything you ever wanted to know about *Saccharomyces cerevisiae* telomeres: beginning to end. *Genetics* 191 1073–1105. 10.1534/genetics.111.137851 22879408 PMC3415994

[B57] Wilkie A. O. Lamb J. Harris P. C. Finney R. D. Higgs D. R. (1990). A truncated human chromosome 16 associated with alpha thalassaemia is stabilized by addition of telomeric repeat (TTAGGG)n. *Nature* 346 868–871. 10.1038/346868a0 1975428

[B58] Xu L. Petreaca R. C. Gasparyan H. J. Vu S. Nugent C. I. (2009). *TEN1* is essential for *CDC13*-mediated telomere capping. *Genetics* 183 793–810. 10.1534/genetics.109.108894 19752213 PMC2778977

[B59] Zhang W. Durocher D. (2010). *De novo* telomere formation is suppressed by the Mec1-dependent inhibition of Cdc13 accumulation at DNA breaks. *Genes Dev.* 24 502–515. 10.1101/gad.1869110 20194442 PMC2827845

[B60] Zhu Z. Chung W.-H. Shim E. Y. Lee S. E. Ira G. (2008). Sgs1 helicase and two nucleases Dna2 and Exo1 resect DNA double-strand break ends. *Cell* 134 981–994. 10.1016/j.cell.2008.08.037 18805091 PMC2662516

